# The psychiatric impact of the 2020 beirut port explosion on civilians and relief workers

**DOI:** 10.1192/j.eurpsy.2021.387

**Published:** 2021-08-13

**Authors:** R. Charara, J. El-Khoury

**Affiliations:** Psychiatry, American University of Beirut Medical Center, Beirut, Lebanon

**Keywords:** ptsd, community mental health, trauma

## Abstract

**Introduction:**

On August 4th 2020, a massive port explosion shook Beirut, killing at least 200, injuring more than 6,000 people and leaving more than a quarter of a million living in unfit homes. Various factors can participate in the severity of mental health outcomes of a disaster including the number of injuries, the degree of property destruction, unexpectedness of the occurrence of the event, and the type of the disaster.

**Objectives:**

The main aim of this study is to assess the prevalence of post-traumatic stress disorder (acute stress disorder) and major depression at 1 and 6 months following the Beirut explosion. The secondary aim is to determine predictors of PTSD incidence among civilians and relief workers affected by the disaster.

**Methods:**

This is a cross-sectional study with data collected via an online survey through convenience sampling. People will be recruited via social media platforms. To achieve a power of 80% and a two-sided significance of 5% and because gender differences will be explored, assuming a design effect (deff) of 2.5, a minimum sample of 960 participants would be needed. The survey will include sociodemographic data, questions about exposure levels to trauma and a psychiatric symptom inventory. Pearson’s Chi Square test will be used to examine the association between categorical variables and regression models will be run to examine the associations while controlling for confounders, including age, gender and others.

**Results:**

The results from both rounds of data collection (months 1 and 6) will be available in late March 2021.

**Conclusions:**

to follow based on results

**Disclosure:**

No significant relationships.

